# Study to Explore the Association of the Renin-Angiotensin System and Right Ventricular Function in Mechanically Ventilated Patients

**DOI:** 10.3390/jcm11154362

**Published:** 2022-07-27

**Authors:** Armand Mekontso Dessap, Kate Hanrott, William M. Powley, Andrew Fowler, Andrew Bayliffe, François Bagate, David A. Hall, Aili L. Lazaar, David C. Budd, Antoine Vieillard-Baron

**Affiliations:** 1 Medical Intensive Care Unit, Henri Mondor Hospital, AP-HP, 94010 Creteil, France; armand.dessap@aphp.fr (A.M.D.); francois.bagate@aphp.fr (F.B.); 2 CARMAS Research Group, UPEC, 94010 Creteil, France; 3 IMRB, INSERM U 955, 94010 Creteil, France; 4 Medicines Research Centre, GlaxoSmithKline plc., Stevenage SG1 2NY, UK; kate_hanrott@hotmail.com (K.H.); william.m.powley@gsk.com (W.M.P.); andrew.bayliffe@gmail.com (A.B.); david.a.hall@gsk.com (D.A.H.); david.c.budd@gsk.com (D.C.B.); 5 GlaxoSmithKline plc., Middlesex TW8 9GS, UK; andrew.x.fowler@gsk.com; 6 Discovery Medicine, Clinical Pharmacology and Experimental Medicine, GlaxoSmithKline plc., Collegeville, PA 19426, USA; aili.l.lazaar@gsk.com; 7 Intensive Care Medicine Unit, Assistance Publique-Hôpitaux de Paris, University Hospital Ambroise Paré, 92100 Boulogne-Billancourt, France; 8 INSERM UMR-1018, CESP, Team Kidney and Heart, University of Versailles Saint-Quentin en Yvelines, 78280 Villejuif, France

**Keywords:** renin-angiotensin system, angiotensin-converting enzyme 2, right ventricular function, acute respiratory distress syndrome, echocardiography, angiotensin II, angiotensin (1–7), pulmonary circulatory dysfunction, acute cor pulmonale

## Abstract

Background: Right ventricular (*RV*) dysfunction is associated with pulmonary vasoconstriction in mechanically ventilated patients. Enhancing the activity of angiotensin-converting enzyme 2 (*ACE*2), a key enzyme of the renin-angiotensin system (*RAS*), using recombinant human *ACE*2 (*rhACE*2) could alleviate *RAS*-mediated vasoconstriction and vascular remodeling. Methods: This prospective observational study investigated the association between concentrations of *RAS* peptides (Ang II or Ang(1–7)) and markers of *RV* function, as assessed by echocardiography (ratio of *RV* to left ventricular end-diastolic area, interventricular septal motion, and pulmonary arterial systolic pressure (*PASP*)). Results: Fifty-seven mechanically ventilated patients were enrolled. Incidence rates of acute cor pulmonale (*ACP*) and pulmonary circulatory dysfunction (*PCD*) were consistent with previous studies. In the 45 evaluable participants, no notable or consistent changes in *RAS* peptides concentration were observed over the observation period, and there was no correlation between Ang II concentration and either *PASP* or *RV* size. The model of the predicted posterior distributions for the pre- and post-dose values of Ang II demonstrated no change in the likelihood of *PCD* after hypothetical dosing with *rhACE*2, thus meeting the futility criteria. Similar results were observed with the other *RAS* peptides evaluated. Conclusions: Pre-defined success criteria for an association between *PCD* and the plasma *RAS* peptides were not met in the mechanically ventilated unselected patients.

## 1. Introduction

The role of the renin-angiotensin system (*RAS*) has been well-described in left ventricular (*LV*) function, but its role in pulmonary circulatory dysfunction (*PCD*) and right ventricular (*RV*) function is not well-known. *RV* dysfunction may result from excessive increases in *RV* preload or afterload or decreased contractility, as a result of injury [1]. *RV* afterload, secondary to increased pulmonary vascular resistance in patients with acute respiratory distress syndrome (*ARDS*) [2], may result in *PCD* [3]. *PCD*, particularly its severe form acute cor pulmonale (*ACP*), has been associated with poor outcomes in critically ill patients, including those with *ARDS* [4,5]. Patients with *ACP* exhibit a higher incidence of shock, increased heart rate, lower arterial pressures, and higher hospital mortality than patients with moderate, or no *PCD* [4].

Angiotensin (Ang) II is a key effector peptide of the *RAS* that drives acute vasoconstriction and vascular remodeling in human diseases, such as pulmonary hypertension [6,7,8,9]. Angiotensin-converting enzyme 2 (*ACE*2) compensates the vasoconstrictive axis of the *RAS* by cleaving Ang II to form Ang(1–7) [10]. Ang(1–7) has demonstrated vasodilatory [11], anti-inflammatory [10,12], anti-fibrotic [10,13], and anti-proliferative effects [12,14,15] in experimental models. Failure of the *RV* is associated with increased activation of the *RAS* in experimental studies [16,17], and increased circulating Ang II concentrations have been observed in humans with *ARDS* [18], thus implicating Ang II in disease pathogenesis.

Current management approaches for acute *RV* failure include efforts to reduce *RV* afterload [1], which is achieved by decreasing pulmonary vascular resistance and increasing pulmonary vasodilation [19]. *RAS*-mediated vasoconstriction and vascular remodeling may be improved through the dual action of *ACE*2 by simultaneously degrading Ang II and forming Ang(1–7). Increasing *ACE*2 activity/concentrations may, therefore, represent a therapeutic strategy for reducing the likelihood of *PCD*. 

This study was planned to investigate the association of *RAS* peptides and markers of *RV* function in participants requiring mechanical ventilation, in order to predict whether the manipulation of the *RAS* by administration of recombinant human *ACE*2 (*rhACE*2) protein would benefit this population. To evaluate the estimated effect of administering *rhACE*2 on *RV* outcomes via its effect on *RAS* peptides, a model was generated, using data from two studies [20,21] to predict the probability of *RV* dysfunction for pre-determined concentrations of *RAS* peptides.

## 2. Methods

### 2.1. Objectives

The primary objective of this study was to evaluate the association between plasma Ang II concentration and *RV* function in mechanically ventilated participants. Secondary objectives were to: (1) define the incidence of *PCD* in this cohort and (2) evaluate the association between plasma Ang(1–7) concentration, Ang II/Ang(1–7) ratio, and *RV* function.

### 2.2. Study Design

**Figure 1:**
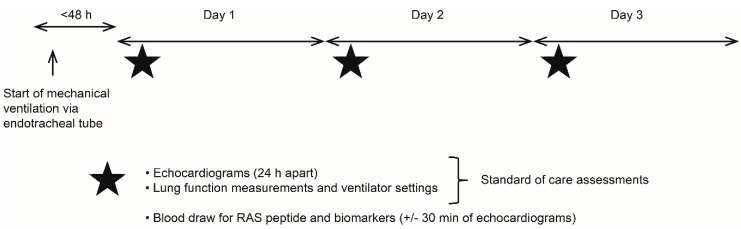
Study design and key study features. *h*, hours; *RAS*, renin-angiotensin system.

This was a prospective, observational, low-interventional study conducted at two centers in France from June 2018 to July 2019 (GlaxoSmithKline plc. study 205821). Participants were enrolled in the study following intubation and mechanical ventilation. Participants were evaluated over a three-day period using standard of care investigations, including transthoracic echocardiography (*TTE*), and/or transesophageal echocardiography (*TOE*). Additional investigations were limited to blood samples for *RAS* peptide analysis (Figure 1). No investigational product was administered. Clinical interventions were based on local standard of care and international guidelines, including fluid resuscitation, use of vasopressor drugs and renal support, and other biomarkers that were not part of standard of care.

### 2.3. Participants

The study population comprised adults aged 18–80 years with a body mass index (*BMI*) of 18.0 to 38.0 kg/m^2^ receiving invasive mechanical ventilation (duration of ventilation ≤48 h) and capable of giving signed informed consent. If participants were not capable of giving signed informed consent, an emergency consent procedure was followed. Participants were excluded from the study if they were moribund, their clinical condition was deteriorating rapidly, or the investigator did not consider there to be a reasonable expectation that the participant would be able to complete the three days of observation in the study. Patients with any of the following were excluded: chronic obstructive pulmonary disease requiring long-term oxygen treatment or home mechanical ventilation, undergoing elective surgery, pre-existing chronic pulmonary hypertension, massive pulmonary embolism or shock, pulmonary vasculitis or pulmonary hemorrhage, lung transplantation in the previous six months, or cardiopulmonary arrest during concurrent illness. Self-reported use of *RAS* modulators, including angiotensin-converting enzyme (*ACE*) type 1 inhibitors, renin inhibitors, and angiotensin receptor blockers, within 4 days or 5.5 half-lives, whichever was longer, and a do-not-resuscitate status, also excluded patients from the study.

### 2.4. Endpoints and Assessments

The primary endpoint was the association between plasma Ang II concentration and *PCD*, as assessed by echocardiography (VIVID E9 and S70 ultrasound system (GEMS, Buc, France)) by measuring ratio of *RV* to *LV* end-diastolic area, interventricular septal motion, and *PASP* [22]). *PCD* was defined as increased pulmonary arterial systolic pressure (*PASP*) > 40 mmHg, increased *RV*/*LV* area ratio >0.6, and/or intraventricular septum dyskinesia. Secondary endpoints were: (1) the presence of *PCD* and its subtypes: moderate *PCD* (*PASP* > 40 mmHg or end-diastolic *RV*/*LV* area ratio ≥ 0.6 without septal dyskinesia), severe *PCD*, i.e., *ACP* (end-diastolic *RV*/*LV* area ratio 0.6 with septal dyskinesia) [5], and severe *ACP* (end-diastolic *RV*/*LV* area ratio ≥1 with septal dyskinesia) [5]; (2) the association between plasma Ang(1–7) concentration, Ang II/Ang(1–7) ratio, and *RV* function, as assessed by echocardiography. The presence of *ARDS*, per the Berlin definition [23], was also assessed as a secondary objective (see Table S1 in Supplementary Materials).

Endpoints were assessed up to and including Day 3 of observation. Participants were assessed by echocardiography within 48 h of mechanical ventilation (Day 1) and every 24 h for a further two days (Days 2 and 3). *TTE* was conducted on all participants, while *TOE* was conducted using a multiplane esophageal probe, when adequate windows could not be obtained using *TTE* (in 17/57 patients). All echocardiographies were performed by trained operators (competent in advanced critical care echocardiography). Images were stored in a digital format on-site, and a computer-assisted consensual interpretation was performed offline by at least two trained senior investigators. The following parameters were used to assess *RV* function: *RV* size (measurement of the end-diastolic *RV*/*LV* area ratio on a 4 chamber-view), interventricular septal motion in the short-axis view of the heart (presence or absence of dyskinesia), and systolic eccentricity index (the ratio of the *LV* short-axis diameter parallel to the septum to the *LV* short-axis diameter perpendicular to the septum) [24]. *PASP* was estimated using tricuspid regurgitation velocity and application of the modified Bernoulli equation [25].

Blood samples for *RAS* peptides were collected at the time of the echocardiogram on each day. Additional parameters (including ventilator settings and arterial blood gases) were collected at the time of intubation and daily, as per standard of care. Daily assessment of mechanical ventilation and mortality continued until hospital discharge or Day 28, whichever was sooner. Safety assessments included serious adverse event collection, safety laboratory assessments, and recording of vital signs. A participant was considered to have completed the study if he/she completed Day 3 assessments.

The evaluable population, defined as all participants for whom *PCD*, Ang II, and Ang(1–7) data were recorded for at least one study time point, was the population used for the primary analyses and majority of the statistical modeling. A population of participants ‘at risk’ of classification for *PCD* and *ARDS*, defined as participants who had a recorded *PCD* and/or *ARDS* assessment during the study period, was used for the secondary analysis of the incidences of *PCD* (and its subtypes) and *ARDS*. The safety population, defined as all participants for whom at least one echocardiograph and/or blood sample was taken, was the population used for all safety analyses.

### 2.5. Statistical Methods

Instead of modeling the association of Ang II concentrations with the dichotomized *RV* dysfunction response variable and losing the information from the continuous variables, a Bayesian repeated measures mixed-effects linear regression model was used to model the association of Ang II concentration with *PCD*. *PASP* and *RV* size ratios were fitted as bivariate outcome variables, with Ang II as the explanatory variable (see additional statistical methods in Supplementary Materials).

The model was used to make a prediction of *PASP* and *RV* size ratio for two reference values of Ang II, i.e., 30 pg/mL and 8 pg/mL. These values were selected as concentrations that could hypothetically occur before and after dosing with *rhACE*2 in this patient population, based on *RAS* peptide analysis by Hemnes et al. [20] and Khan et al. [21]. An estimate for the probability of *PCD* was derived for each of the given Ang II concentrations. The difference in predicted probabilities was used to assess whether there was sufficient evidence for an association between *PCD* and Ang II using a priori decision criteria. A percentage point difference >30 between the probabilities was considered successful, percentage point differences between 15 and 30 were considered uncertain, and a percentage point difference <15 was deemed futile. It was proposed that a *PCD* risk reduction of at least 30%, due to therapeutic intervention with *rhACE*2, is a large enough effect size to have a reasonable chance of translating to a detectable mortality improvement in future pivotal studies. 

The secondary analyses of the association of Ang(1–7) concentration with *PCD* and of Ang II/Ang(1–7) concentrations with *PCD* were both modelled per the primary analysis. Hypothetical ‘pre-dose’ and ‘post-dose’ reference values for Ang(1–7) concentrations were 2 and 30 pg/mL, respectively, and based on the *RAS* peptide analysis from the same studies [20,21] as for the Ang II concentrations. 

The planned overall sample size of 150 participants was chosen to provide a precision of ±5% points (defined as the half-width of the 95% Wald confidence interval [CI] [26]) around a point estimate for *ACP* incidence (based on an estimated incidence rate of 10%). However, it was possible to conduct the primary analysis (association between Ang II and *PCD*, measured by *PASP* and *RV* size ratio) without reaching the target precision of ±5% points, when ~50 participants had evaluable data. Further information on statistical methods can be found in the Supplementary Materials.

## 3. Results

### 3.1. Study Population

**Figure 2:**
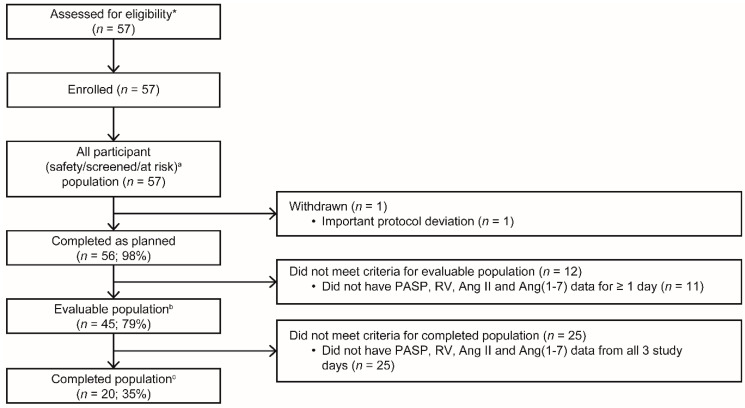
Participant flow. * Patients were pre-screened for eligibility; however, the number of pre-screened patients is unavailable. ^a^ Screened population: all participants who were screened. Safety population: all participants for whom at least one echocardiograph and/or blood sample was taken. At-risk population: participants for whom *PASP* and *RV* size ratio were recorded for all three study days and/or had a databased *PCD* and/or *ARDS* assessment during the study period. ^b^ Evaluable population: all participants for whom *PASP*, *RV* size ratio, Ang II, and Ang(1–7) data were recorded for at least one study time point. ^c^ Completed population: all participants for whom *PASP*, *RV* size ratio, Ang II, and Ang(1–7) data were recorded for all three study days. *ACP*, acute cor pulmonale; *Ang*, angiotensin; *ARDS*, acute respiratory distress syndrome; *PASP*, pulmonary arterial systolic pressure; *PCD*, pulmonary circulatory dysfunction; *RV*, right ventricle.

During study progression, *rhACE*2 development was terminated, leading to early study close. A total of 57 participants were enrolled; there were no screen failures (Figure 2). One participant was withdrawn, due to a protocol deviation (violation of the inclusion criteria for *BMI* and age). Demographics and clinical characteristics of the participants were similar between patients with and without *PCD* (Table 1). Most participants (*n* = 40/57; 70%) were managed with vasopressors, inotropes, and other vasoactive agents at baseline. The most common reasons for intubation were acute respiratory failure (*n* = 25/57; 44%), followed by sepsis (*n* = 10/57; 18%) and impaired neurological status or post-surgical management (*n* = 16/57; 28%). Baseline echocardiography did not reveal significant chronic *RV* dysfunction. Respiratory variables and catecholamine support at each visit are reported in  Supplemental Tables S2 and S3, respectively, in Supplemental Material.

### 3.2. Association between Plasma Ang II Concentration and PCD

**Figure 3:**
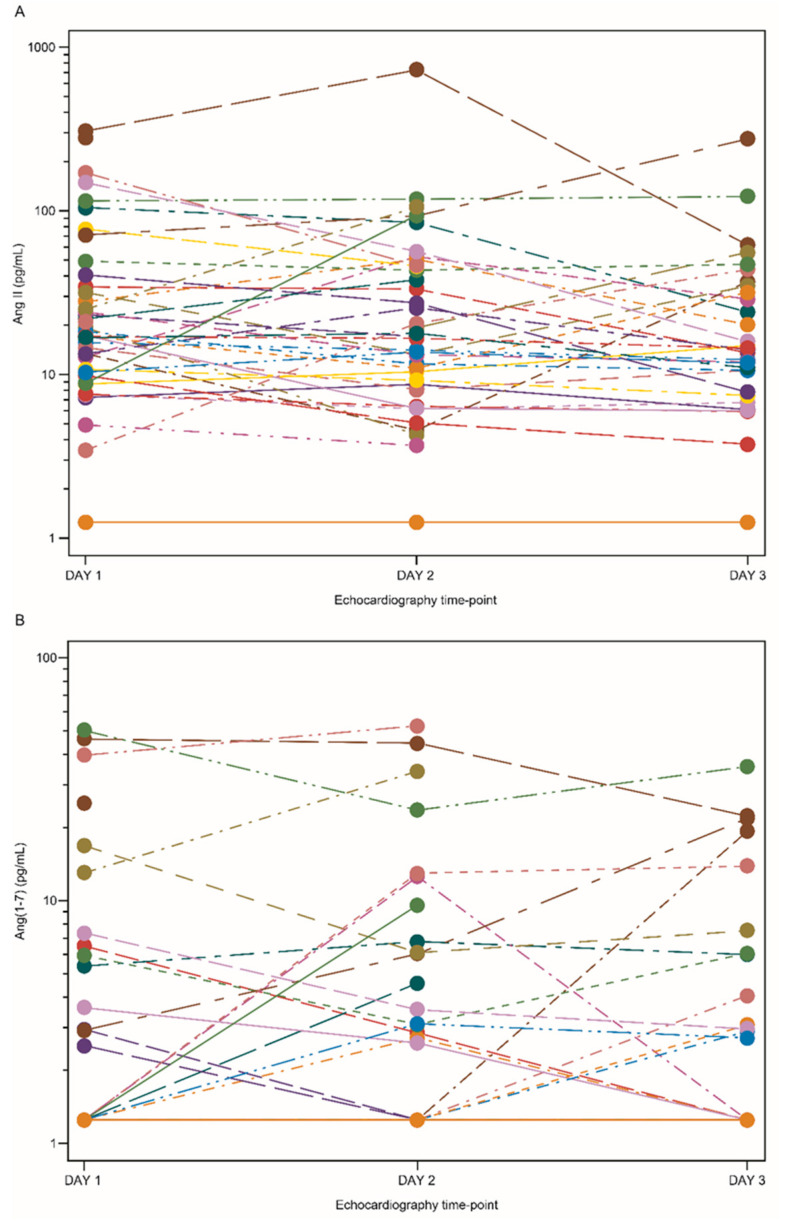
Individual time profile of (**A**) Ang II and (**B**) Ang(1–7) concentrations by time point. Ang angiotensin.

**Figure 4:**
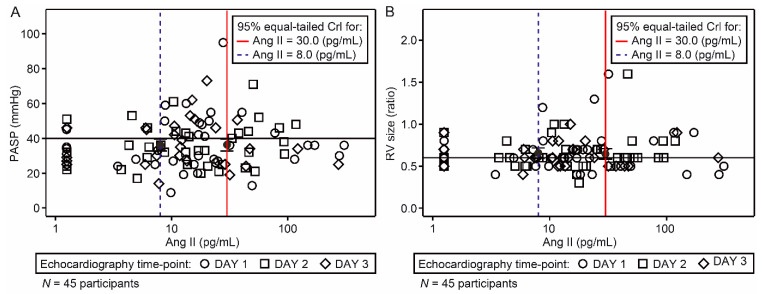
Scatterplots of *PASP* (**A**) and *RV* size ratio (**B**) versus Ang II concentrations, with posterior prediction regions for *RV* function. Data are median *PASP* and *RV* size and 95% equal-tailed credibility intervals for the corresponding ‘pre-dose’ 30 pg/mL and ‘post-dose’ 8 pg/mL Ang II. One measurement was databased per day, per participant. The horizontal reference line corresponds to the defined threshold value associated with *PCD*. *Ang*, angiotensin; *CrI*, credible interval; *PASP*, pulmonary arterial systolic pressure; *RV*, right ventricular.

**Figure 5:**
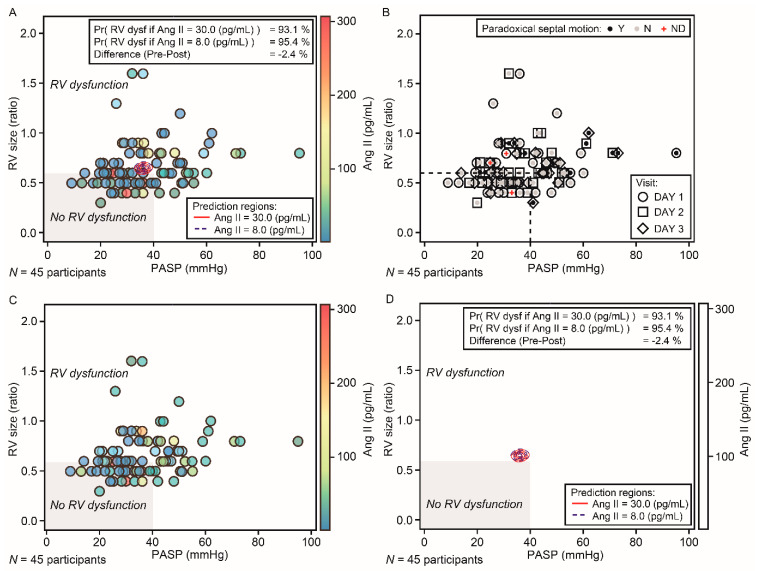
*PCD* measurements for hypothetical ‘pre-‘ and ‘post-‘dose Ang II concentrations (**A**); scatterplot of *PASP* versus *RV* size ratio, marked by interventricular septal motion status (**B**) and colored by Ang II concentration (**C**), with posterior prediction regions (**D**). Data are for ‘pre-dose’ 30 pg/mL and ‘post-dose’ 8 pg/mL Ang II concentrations. The horizontal reference lines correspond to the defined threshold value for *PCD*. Figure 4A contains additional sampling points that did not have an associated Ang II concentration value. *Ang*, angiotensin; *N*, no; *ND*, not done; *PASP*, pulmonary arterial systolic pressure; *PCD*, pulmonary circulatory dysfunction; *Pr*, probability; *Y*, yes.

A total of 45 participants were included in the evaluable population for the primary analysis. Although the individual time-profile plots of Ang II demonstrated that most participants had Ang II concentrations above those seen in healthy participants (physiological concentrations of 8 pg/mL [27]), there were no notable or consistent changes in Ang II concentration over time (Figure 3). Individual *PASP* and *RV* model predictions were similar at the hypothetical Ang II concentrations before and after ‘dosing’ with *rhACE*2 (30 pg/mL and 8 pg/mL, respectively) (Figure 4). The probability of *PCD*, given an Ang II concentration of 30 pg/mL (hypothetical ‘pre-dose’ value), was estimated to be 93.1%; the probability of *PCD*, given an Ang II concentration of 8 pg/mL (hypothetical ‘post-dose’ value), was estimated to be 95.4% (Figure 5A). As regression gradients were flat, the difference (−2.4%) in predicted probability was likely an artefact of noise. The difference was compared with the pre-defined criteria. It did not exceed 15% and, therefore, met the futility criterion. The observed patient-level data of *PASP* versus *RV* size ratio, marked by interventricular septal motion status, are shown in Figure 5B. The statistical model was used to predict the probability of *PCD* for a given concentration of Ang II (Figure 5C). Subsequently, the model-predicted posterior distributions for the hypothetical ‘pre-dose’ and ‘post-dose’ concentrations of Ang II were generated, in which a large overlap between the distributions was observed (Figure 5D).

### 3.3. Association between Plasma Ang(1–7) Concentration, Ang II/Ang(1–7) Ratio, and PCD

**Figure 6:**
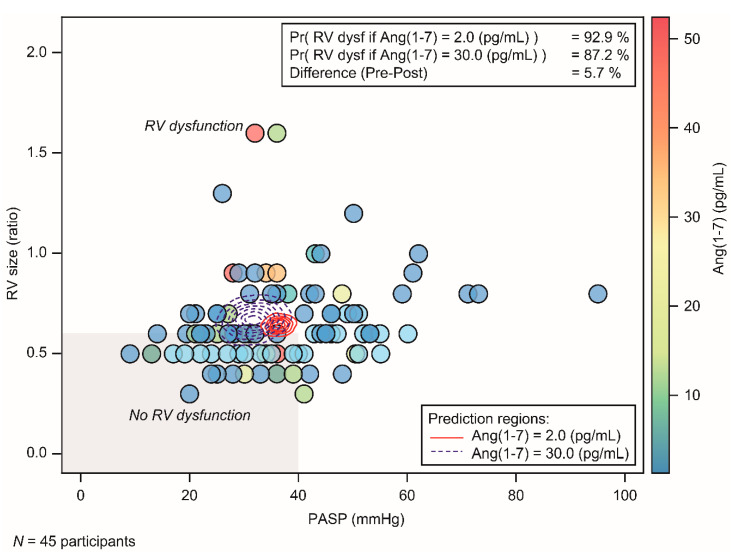
Scatterplot of *PASP* versus *RV* size ratio, marked by interventricular septal motion status and colored by Ang(1–7) concentration, with posterior prediction regions for *PCD* measurements for hypothetical ‘pre-‘ and ‘post-‘dose Ang(1–7) concentrations. Data are for ‘pre-dose’ 2 pg/mL and ‘post-dose’ 30 pg/mL Ang II concentrations. The horizontal reference lines correspond to the defined threshold value for *PCD*. *Ang*, angiotensin; *PASP*, pulmonary arterial systolic pressure; *Pr*, probability; *RV*, right ventricular.

Data from the 45 participants in the evaluable population were used for this analysis. Consistent with the Ang II results, the time profile of the Ang(1–7) and Ang II/Ang(1–7) ratio showed no notable or consistent changes in Ang(1–7) concentration or Ang II/Ang(1–7) ratio over the study period (Figure 3 and Figure S1 in Supplementary Materials). The model predicted that increasing Ang(1–7) from 2 to 30 pg/mL would reduce the probability of *PCD* by ~5.7 percentage points (Figure 6). Although there was a supportive trend towards a decreased likelihood of dysfunction with increased Ang(1–7) concentration, the difference in predicted probabilities between pre- and post-dose did not exceed 15% and, thus, met the futility criterion.

### 3.4. Incidence of PCD

The incidence rates of *PCD* and its subtypes (including *ACP* and severe *ACP*) were measurable in 57 participants; therefore, data from all 57 participants were used in this secondary analysis. A summary of *PCD* subtypes and *ARDS* incidence rates is presented in Table 2.

Overall, 51% of patients had *PCD* (*n* = 29/57 (95% CI 37, 64)). A total of 21% of patients had *ACP* (*n* = 12/57 (95% CI 12, 34)), and few patients had severe *ACP* (*n* = 3/57; 5% (95% CI 1, 16)). Approximately one-quarter of the participants had a diagnosis of *ARDS* (*n* = 15/57; 26% (95% CI 16, 40)).

Safety data are summarized in the Supplementary Materials.

## 4. Discussion

Few studies have evaluated the association between the *RAS* and *PCD* in humans. The objective of this study was to investigate the association of *RAS* peptides and markers of *PCD* in participants requiring mechanical ventilation, as well as to further characterize this patient population. The *PCD* incidence rates seen in this study were in line with those observed previously in a similar population [5]. Overall, we observed no consistent changes in Ang II or Ang(1–7) concentration over the observation period. Despite a significant range of Ang II concentrations, indicating the *RAS* was activated in many patients, the results did not demonstrate a relationship between Ang II concentrations and either *PASP* or *RV* size. The same conclusions applied to Ang(1–7).

It is interesting to compare our results to the ones published by Khan et al. in patients with moderate *ARDS* [21]. While our population of 45 mechanically ventilated patients is quite different and more heterogeneous, with only 26% of *ARDS*, we finally found results similar to the 20 patients randomized in their control group. AngII was indeed significantly elevated, had a large distribution of values, and little change over the first days following the inclusion, as was also reported by Reddy et al. [18]. This could suggest that more than the *ARDS*, per se, it is the need for mechanical ventilation that mainly induces *RAS* peptides alterations. 

Most participants had Ang II concentrations above those seen in healthy participants, and no substantial changes in Ang II concentration over time were observed. A previous study of patients with heart failure demonstrated that, despite *ACE* inhibitor therapy, Ang II concentrations remained persistently higher than those seen in healthy individuals in half of the study population [28]. Experimental studies suggest that Ang II plays an important role in ventricular remodeling [28]. Ang II may provide a positive feedback loop in vascular remodeling by upregulating the expression of pro-fibrotic factors, thus resulting in further production of Ang II, therefore perpetuating vascular remodeling [29].

A Bayesian repeated measures mixed-effects regression model was used to explore the association of Ang II with *PCD*, based on prediction of *PASP* and *RV* size ratio, according to hypothetical Ang-II concentrations pre- (30 pg/mL) and post- (8 pg/mL) dosing with *rhACE*2. If high Ang II concentrations were associated with *PCD* and/or low Ang II concentrations were associated with no *PCD*, this would provide a supportive rationale for the use of *rhACE*2 as a potential treatment in this patient population, as lowering Ang II concentrations with *rhACE*2 could reduce the likelihood of *PCD*. The model of predicted posterior distributions for the pre- and post-dose values showed a large overlap between the distributions, thus indicating no change in the likelihood of *PCD* as Ang II concentrations decreased from 30 to 8 pg/mL. These data suggest that a reduction in Ang II concentrations after treatment with *rhACE*2 would not have a clinically significant impact on *PCD* in this population. The probability of *PCD*, given an Ang II concentration at the hypothetical ‘pre-dose’ value, was high and similar to the ‘post-dose’ value. The difference between the predicted probabilities met the futility criterion, indicating that a future study to evaluate *rhACE*2 as a treatment in patients with severe respiratory failure would likely be unsuccessful. The high probability of *PCD* at the post-dose Ang II concentration was surprising. These results could suggest that the association of *RAS* peptide levels with *PCD* may have been confounded by the impact of mechanical ventilation, in particular the use of high positive end-expiratory pressure on *RAS* peptide concentrations. In fact, transpulmonary pressure may have a direct effect on the compression of pulmonary vessels [30]. Other factors may also be involved in *PCD* during *ARDS*, including hypercapnia [31,32]. Therapeutic dosing of Ang II has recently been evaluated as a vasopressor treatment for distributive shock, with evidence of efficacy [33] that led to approval by the US Food and Drug Administration [34], further highlighting the complex interaction between the *RAS* and both systemic and pulmonary vascular function. Most mechanically ventilated patients were ventilated for acute respiratory failure, and mechanical ventilation itself may have induced *PCD* in the study population, especially when associated with low partial pressure of oxygen (*PaO*_2_). Similar results were observed with the other *RAS* peptides evaluated; no association between the *PCD* and Ang (1-7) concentrations or Ang II/Ang (1-7) ratio was observed.

Consistent with our study, a systematic review on the effects of *ACE* inhibitors, Ang II receptor blockers, and aldosterone antagonists in adults with congenital heart disease and *RV* dysfunction found that *RAS* inhibition did not have a beneficial effect in these patients, suggesting a lack of association between *RAS* peptides and *RV* function. However, the studies included in the review generally had low patient numbers, short follow-up periods, and evaluated surrogate endpoints [16]. Clinical studies of *rhACE*2 in patients with pulmonary arterial hypertension have demonstrated rapid modulation of *RAS* peptides but inconsistent changes in clinical outcomes, including pulmonary hemodynamics [35]. Similarly, in a meta-analysis investigating the role of *RAS* inhibition on *RV* function, the treatment arm (in which patients were treated with *ACE* inhibitors and/or angiotensin receptor blockers) showed a trend towards increased *RV* ejection fraction, compared with the control arm, suggesting a potential role of the *RAS* pathway in *RV* dysfunction. However, as with our present study, the results were not significant, indicating that further, larger prospective studies are needed to further elucidate the role of the *RAS* in *RV* function [13].

### Strengths and Limitations

We conducted this prospective, observational study with low patient intervention to determine whether an investigational product could benefit a patient population. Without exposing patients to a potentially non-beneficial investigational drug, we conceptually determined that an interventional study would not be successful. A broad population of mechanically ventilated participants in critical care were enrolled, increasing the potential generalizability of the study results. However, the number of pre-screened patients was unavailable, and investigating particular patient subgroups may have yielded more specific results on the role of *RAS* peptides in the pathophysiology of severe respiratory failure based on specific diagnoses. It is possible the heterogeneity of the patient population confounded the ability to observe a detectable relationship between the *RAS* peptide levels and *PCD*, as *RAS* is known to be influenced by physiological characteristics, such as *BMI*, vascular tone, renal function, and by co-medications. COVID-19-related *ARDS* may be a relevant target population, given the burden of lung vascular dysfunction in this subgroup [36,37]. This was a small double-centre study, which may limit the generalizability of the findings. The study was limited in its feasibility; obtaining all data from each patient was challenging. As 21 patients were excluded for missing data (because *PCD*, Ang II, and Ang(1–7) data were not available for at least one study time point), this may have induced selection bias in the results and precludes any definite conclusion. A further limitation was that *PASP* was estimated by echocardiography as a surrogate measure, rather than as a direct measurement using invasive measures, which is more robust than echocardiography.

## 5. Conclusions

The results of this study did not produce sufficient evidence to meet pre-defined success criteria regarding evidence for an association between *PCD* in mechanically ventilated patients and plasma *RAS* peptides Ang II, Ang(1–7), or Ang II/Ang(1–7) ratio. Further studies of specific patient subgroups (such as patients with *ARDS* and patients with a high-risk score for developing *ACP*) could help further elucidate the role of *RAS* peptides in these patients.

## Figures and Tables

**Table 1:** Participant demographics and clinical characteristics at inclusion.

Demographics	Total (*n* = 57)	No *PCD* (*n* = 28)	Any *PCD* (*n* = 29)
Age, years (mean (*SD*)) *	63.7 (12.8)	64.3 (13.3)	63.2 (12.5)
Male (*n* (%))	33 (58)	15 (54)	18 (62)
*BMI*, kg/m^2^ (mean (*SD*))	24.8 (5.2)	24.3 (5.2)	25.2 (5.1)
Height, cm (mean (*SD*))	169 (10)	170 (10)	168 (9)
Weight, kg (mean (*SD*))	71 (16)	71 (18)	71 (15)
Clinical characteristics			
Total *SOFA* component score (excluding *CNS*) (mean (*SD*))	7.8 (3.7)	7.5 (3.6)	8.1 (3.9)
Managed with vasopressors, inotropes and other vasoactive agents (*n* (%))			
Yes	40 (70)	19 (68)	21 (72)
No	17 (30)	9 (32)	8 (28)
*PaO*_2_/*FiO*_2_ (mean (*SD*))	208 (103)	223 (113)	193 (92)
*SAPS* II at screening	53 (21)	59 (19)	48 (21)
Reason for intubation (*n* (%))			
Acute respiratory failure	25 (44)	11 (39)	14 (48)
Sepsis	10 (18)	7 (25)	3 (10)
Impaired neurological status or post-surgery management	16 (28)	6 (21)	10 (34)
Low *GCS*	2 (4)	0	2 (7)
No reason for intubation supplied	4 (7)	4 (14)	0
Tidal volume, mL (mean (*SD*))	434 (112)	441 (141)	427 (79)
*PEEP*, cm *H*_2_*O* (mean (*SD*))	6 (4)	6 (4)	7 (4)
Plateau pressure, cm *H*_2_*O* (mean (*SD*))	18 (5)	16 (3)	19 (5)

* Age was imputed when full date of birth was not provided. *BMI*, body mass index; *cm H*_2_*O*, centimeters of water; *CNS*, central nervous system; *FiO*_2_, fraction of inspired oxygen; *GCS*, Glasgow coma scale; *PaO*_2_, partial pressure of oxygen; *PCD*, pulmonary circulatory dysfunction; *PEEP*, positive end-expiratory pressure; *SAPS*, simplified acute physiology score; *SOFA*, sequential organ failure assessment; *SD*, standard deviation.

**Table 2:** Summary of *PCD* and *ARDS* incidence rates.

Disease Status	Frequency, *n* (*N* = 57)	Rate, % (95% CI)
No *PCD* or *ACP*	28	49 (36, 63)
Any *PCD* or *ACP*	29	51 (37, 64)
*PCD*	14	25 (15, 38)
*ACP*	12	21 (12, 34)
Severe *ACP*	3	5 (1, 16)
*ARDS*	15	26 (16, 40)
*ARDS* and no *PCD* or *ACP*	5	9 (3, 20)
*ARDS* and *PCD*	4	7 (2, 18)
*ARDS* and *ACP*	6	11 (4, 22)

*N*: number of participants with ≥1 completed disease status assessment (*ARDS* or *PCD*). Frequency: number of participants with ≥1 disease status answer of ‘yes’, where, for the combined disease status, categories of each of the relevant disease assessments need to be positive ≥ once across visits. Rate: (Frequency/*N*) ∗ 100, 95% CI method: Wilson score interval with continuity correction. *ACP*, acute cor pulmonale; *ARDS*, acute respiratory distress syndrome; *CI*, confidence interval; *PCD*, pulmonary circulatory dysfunction.

## Data Availability

The anonymized aggregate data and study protocol documents from this study are available upon request from the authors, pending approval from GlaxoSmithKline plc.

## Supplementary Materials

The following supporting information can be downloaded at: www.mdpi.com/article/10.3390/jcm11154362/s1, Supplementary materials: Table S1. Disease diagnoses; Additional statistical methods; Tables S2. Patient respiratory profile at different time points; Table S3. Catecholamine support at different time points; Supplemental Figure S1. Individual time profile of Ang II/Ang(1–7) ratio by time point.

## Abbreviations

*ACE*, angiotensin-converting enzyme; *ACE*2, angiotensin-converting enzyme 2; *ACP*, acute cor pulmonale; *Ang*, angiotensin; *ARDS*, acute respiratory distress syndrome; *BMI*, body mass index; *CI*, confidence interval; *cm H_2_O*, centimeters of water; *CNS*, central nervous system; *FiO*_2_, fraction of inspired oxygen; *GCS*, Glasgow coma scale; *h*, hours; *LV*, left ventricular; *PaO*_2_, partial pressure of oxygen; *PASP*, pulmonary arterial systolic pressure; *PCD*, pulmonary circulatory dysfunction; *PEEP*, positive end-expiratory pressure; *RAS*, renin-angiotensin system; *rhACE*2, recombinant human *ACE*2; *RV*, right ventricular; *SAPS*, simplified acute physiology score; *SD*, standard deviation; *SOFA*, sequential organ failure assessment; *TOE*, transesophageal echocardiography; *TTE*, transthoracic echocardiography.
