# Supplementary material for: Study to Explore the Association of the Renin-Angiotensin System and Right Ventricular Function in Mechanically Ventilated Patients
Source: J Clin Med. 2022 Jul 27;11(15):4362. doi: 10.3390/jcm11154362 (PMC9369375; doi:10.3390/jcm11154362)
Supplement: Supplementary file 1 [file jcm-11-04362-s001.zip › jcm-1744689-supplementary.pdf]

Supplementary material

Study to explore the association of the renin-angiotensin system and right ventricular function in mechanically ventilated patients

Armand Mekontso Dessap, Kate Hanrott, William M. Powley, Andrew Fowler, Andrew Bayliffe, François Bagate, David A. Hall, Aili L. Lazaar, David C. Budd, Antoine Vieillard Baron

Additional statistical methods

Conditional on the supplied Ang II concentration, the output from the model is a joint posterior distribution for PASP and RV size ratio values representing plausible combinations that may be observed at the supplied Ang II concentration. The definition of PCD (PASP > 40mmHg or RV/LV area ratio > 0.6) defines a region, and the proportion of the posterior distribution overlapping this region estimates the probability of PCD for the supplied Ang II concentration. The complex correlation structure of the design (correlations between the two endpoints and the repeated measures of each endpoint) was modeled using separate and smaller

variance covariance structures (2x2 unstructured for the endpoints and an 3x3 AR(1) for the repeated measures). These were combined using a Kronecker product. This improved the convergence properties of the model (fewer parameters to estimate) and allowed the “nuisance” parameter of time to be eliminated from the subsequent predictions. The joint posterior distribution is influenced by the individual relationships between Log(Ang II) versus PASP and Log(Ang II) versus RV size ratio because they determine the x- and y-axis co-ordinates of the joint posterior distribution central point in the region (each endpoint controls a particular axis). For example, in the hypothetical situation where there is a strong positive linear relationship between Log(Ang II) and PASP (steep slope) but no relationship between Log(Ang II) and RV size ratio (flat slope), the predictions for low and high Ang II concentrations would be different for PASP, but similar for RV size ratio. This would lead to a posterior distribution that appears to shift only along the direction of the x-axis, potentially changing the proportion of the distribution overlapping with the PCD region for large shifts. The size of the observed shift depends on the magnitude of the slope parameter and the pair of Ang II concentrations selected for the prediction. Strong associations between Ang II, PASP and RV size ratio are expected to manifest in non-zero slopes for both underlying linear relationships and non-overlapping joint posterior distributions when using a reasonably spaced pair of Ang II concentrations.

Safety

The safety population of 57 participants was used for all safety analyses. No serious adverse events were reported. Laboratory values and vital sign abnormalities were consistent with a critically ill population.

Supplemental Table S1. Disease diagnoses

**Disease**	**Diagnostic criteria**
ACP	Dilated RV in the mid-esophagus longitudinal view or apical four-chamber view (end-diastolic RV/LV area ratio [0.6]) associated with the presence of septal dyskinesia in the (transgastric) short-axis view of the heart
Severe ACP	Severely dilated RV (end-diastolic RV/LV area ratio ≥ 1) with septal dyskinesia. Septal dyskinesia will be particularly sought at end-systole, while analyzing loops in slow motion

ARDS[a]	Timing: Within 1 week of a known clinical insult or new or worsening respiratory symptoms<br>Chest imaging: Bilateral opacities not fully explained by effusions, lobar/lung collapse or nodules<br>Origin of oedema: Respiratory failure not fully explained by cardiac failure or fluid overload. Need objective assessment (e.g., echocardiography) to exclude hydrostatic oedema if no risk factor present<br>Oxygenation:<br>• Mild: 200 mm Hg $< PaO_2/FiO_2 \leq$ 300 mm Hg with PEEP $\geq$ 5 cm H_2O<br>• Moderate: 100 mm Hg $< PaO_2/FiO_2 \leq$ 200 mm Hg with PEEP $\geq$ 5 cm H_2O<br>• Severe: $PaO_2/FiO_2 \leq$ 100 mm Hg with PEEP $\geq$ 5 cm H_2O

[a]Berlin definition.

ACP acute cor pulmonale; *ARDS* acute respiratory distress syndrome; *cm* H_2O centimeters of water; FiO_2 fraction of inspired oxygen; *LV* left ventricle; PaO_2 partial pressure of oxygen; *PEEP* positive end-expiratory pressure; *RV* right ventricular.

Supplemental Table S2. Patient respiratory profile at different time points

Variable		N	Visit	n	Mean	SD	Median	Min.	Max.
PEEP (cm H_2O)	Any PCD/ACP	29	DAY 1	29	6.966	4.2214	5.000	0.00	20.00
			DAY 2	24	6.708	3.5322	5.000	0.00	15.00
			DAY 3	21	6.810	2.7133	5.000	5.00	12.00
	No PCD/ACP	28	DAY 1	27	5.852	4.4177	5.000	0.00	25.00
			DAY 2	24	5.667	1.5228	5.000	5.00	10.00
			DAY 3	18	5.722	2.1367	5.000	0.00	10.00
	All Participants	57	DAY 1	56	6.429	4.3143	5.000	0.00	25.00
			DAY 2	48	6.188	2.7418	5.000	0.00	15.00
			DAY 3	39	6.308	2.4937	5.000	0.00	12.00
Plateau pressure (cm H_2O)	Any PCD/ACP	29	DAY 1	22	19.227	4.9755	18.000	12.00	31.00
			DAY 2	12	19.583	4.8516	18.500	14.00	27.00
			DAY 3	12	19.250	4.1588	18.000	15.00	26.00
	No PCD/ACP	28	DAY 1	22	15.909	3.5309	15.500	12.00	27.00
			DAY 2	15	16.867	4.9838	16.000	10.00	28.00
			DAY 3	11	16.818	4.6652	18.000	10.00	25.00
	All Participants	57	DAY 1	44	17.568	4.5821	16.500	12.00	31.00
			DAY 2	27	18.074	5.0225	18.000	10.00	28.00
			DAY 3	23	18.087	4.4814	18.000	10.00	26.00
Tidal Volume (mL)	Any PCD/ACP	29	DAY 1	29	427.414	78.5882	440.000	280.00	650.00
			DAY 2	23	413.696	84.4799	390.000	280.00	590.00
			DAY 3	19	423.526	84.7259	400.000	280.00	560.00
	No PCD/ACP	28	DAY 1	27	441.407	141.0263	455.000	5.00	640.00
			DAY 2	24	509.917	92.3801	500.000	380.00	750.00

			DAY 3	18	527.722	148.7109	500.000	363.00	1000.00
	All Participants	57	DAY 1	56	434.161	112.2309	450.000	5.00	650.00
			DAY 2	47	462.830	100.2207	460.000	280.00	750.00
			DAY 3	37	474.216	129.6918	460.000	280.00	1000.00
PaO2/FiO2 ratio	Any PCD/ACP	29	DAY 1	29	193.407	92.1688	195.000	45.00	440.00
			DAY 2	29	191.390	76.0851	188.000	59.00	344.30
			DAY 3	25	232.280	103.4942	208.000	93.00	468.00
	No PCD/ACP	28	DAY 1	28	223.286	112.8486	213.000	30.00	460.00
			DAY 2	25	237.412	118.6584	243.000	20.00	440.00
			DAY 3	22	241.673	133.7419	220.000	30.00	556.00
	All Participants	57	DAY 1	57	208.084	103.0274	200.000	30.00	460.00
			DAY 2	54	212.696	99.8530	220.000	20.00	440.00
			DAY 3	47	236.677	117.3736	210.000	30.00	556.00
Respiration rate (breaths/min)	Any PCD/ACP	29	DAY 1	29	23.724	6.9277	25.000	12.00	35.00
			DAY 2	27	24.889	6.1603	25.000	15.00	35.00
			DAY 3	26	24.769	7.4098	25.000	12.00	38.00
	No PCD/ACP	28	DAY 1	24	17.042	5.3200	15.000	8.00	30.00
			DAY 2	21	18.048	4.6312	17.000	12.00	30.00
			DAY 3	21	17.048	4.9343	16.000	10.00	26.00
	All Participants	57	DAY 1	53	20.698	7.0454	20.000	8.00	35.00
			DAY 2	48	21.896	6.4717	21.000	12.00	35.00
			DAY 3	47	21.319	7.4487	20.000	10.00	38.00

Mean Airway Pressure (cmH2O)	Any PCD/ACP	29	DAY 1	20	12.395	5.3221	10.500	4.70	26.00
			DAY 2	19	11.611	4.4451	9.000	8.00	22.00
			DAY 3	14	16.464	18.6491	9.500	8.00	80.00
	No PCD/ACP	28	DAY 1	16	9.350	3.0018	8.800	6.00	18.00
			DAY 2	15	14.020	17.1233	9.000	5.00	75.00
			DAY 3	7	17.457	22.0278	8.400	6.00	67.00
	All Participants	57	DAY 1	36	11.042	4.6468	9.300	4.70	26.00
			DAY 2	34	12.674	11.6894	9.000	5.00	75.00
			DAY 3	21	16.795	19.2836	9.000	6.00	80.00

Supplemental Table S3. Catecholamines at different time points

Visit		Catecholamines	Any PCD/ACP (N=29)		No PCD/ACP (N=28)		All (N=57)	
Day	1	n	21		19		40	
		NOREPINEPHRINE	21	(72.4%)	19	(67.9%)	40	(70.2%)
		DOBUTAMINE	0		1	(3.6%)	1	(1.8%)
Day	2	n	15		16		31	
		NOREPINEPHRINE	15	(51.7%)	15	(53.6%)	30	(52.6%)
		DOBUTAMINE	0		1	(3.6%)	1	(1.8%)
Day	3	n	10		7		17	
		NOREPINEPHRINE	10	(34.5%)	7	(25.0%)	17	(29.8%)
		DOBUTAMINE	0		0		0	

Supplemental Figure S1. Individual time profile of Ang II/Ang(1-7) ratio by time point

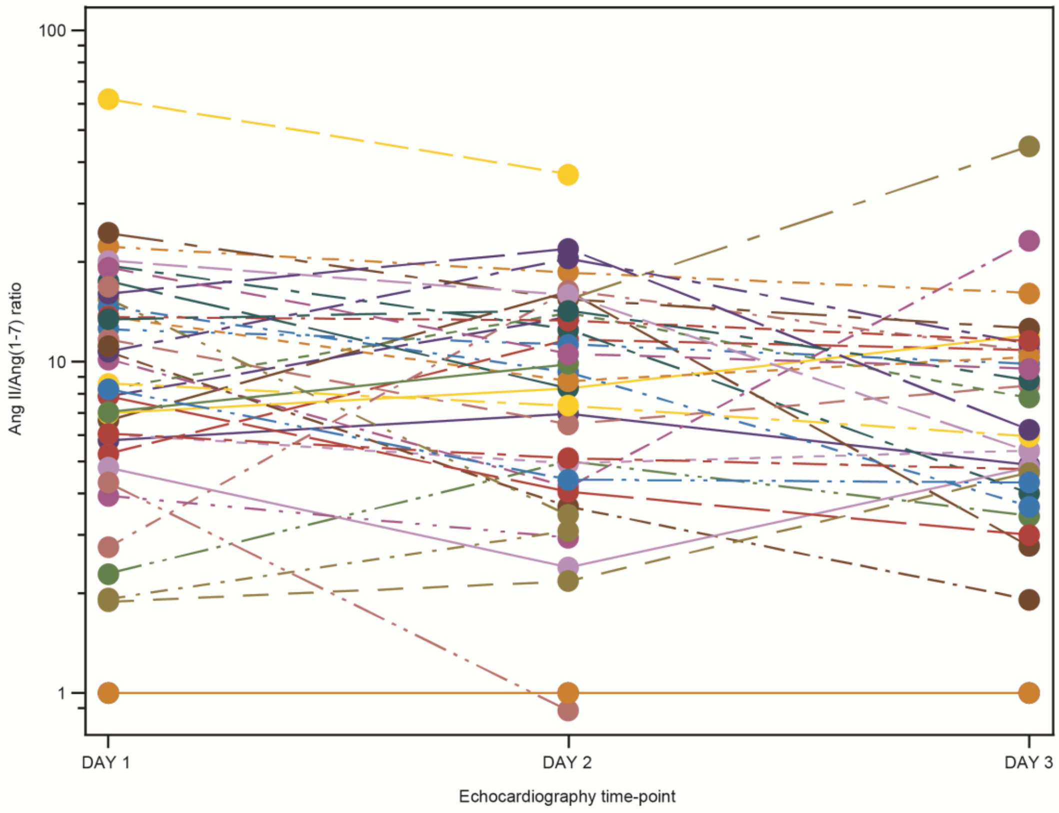

Ang angiotensin.
